# Southward re‐distribution of tropical tuna fisheries activity can be explained by technological and management change

**DOI:** 10.1111/faf.12443

**Published:** 2020-01-28

**Authors:** Iratxe Rubio, Unai Ganzedo, Alistair J. Hobday, Elena Ojea

**Affiliations:** ^1^ Basque Centre for Climate Change (BC3) Leioa Spain; ^2^ Future Oceans Lab University of Vigo Vigo Spain; ^3^ Marine Support Derio Spain; ^4^ CSIRO Oceans and Atmosphere Hobart Tas Australia; ^5^ Centre for Marine Socioecology University of Tasmania Hobart Tas Australia

**Keywords:** bilateral agreements, effort, fisheries management, ocean warming, technology

## Abstract

There is broad evidence of climate change causing shifts in fish distribution worldwide, but less is known about the response of fisheries to these changes. Responses to climate‐driven shifts in a fishery may be constrained by existing management or institutional arrangements and technological settings. In order to understand how fisheries are responding to ocean warming, we investigate purse seine fleets targeting tropical tunas in the east Atlantic Ocean using effort and sea surface temperature anomaly (SSTA) data from 1991 to 2017. An analysis of the spatial change in effort using a centre of gravity approach and empirical orthogonal functions is used to assess the spatiotemporal changes in effort anomalies and investigate links to SSTA. Both analyses indicate that effort shifts southward from the equator, while no clear pattern is seen northward from the equator. Random forest models show that while technology and institutional settings better explain total effort, SSTA is playing a role when explaining the spatiotemporal changes of effort, together with management and international agreements. These results show the potential of management to minimize the impacts of climate change on fisheries activity. Our results provide guidance for improved understanding about how climate, management and governance interact in tropical tuna fisheries, with methods that are replicable and transferable. Future actions should take into account all these elements in order to plan successful adaptation.

## 1 INTRODUCTION

Fisheries are expected to satisfy an increasing proportion of global protein demand in the future, but can only do this if managed sustainably (Barange et al., 2018; Merino et al., 2012). This is a considerable challenge given human population growth and the projected increase in food demand (Tilman & Clark, 2014). Wild fisheries support industrial and traditional livelihoods worldwide, from domestic subsistence artisanal fishing (Golden et al., 2016), to highly technological industrial fleets operating across jurisdictional boundaries (Lopez, Moreno, Sancristobal, & Murua, 2014). Managing international fisheries sustainably is difficult given the complexity of marine governance, exclusive economic zones (EEZs) and international agreements (Miller, 2007). Climate change is posing an additional burden on fisheries management by affecting abundance, phenology and causing distribution shifts of fish stocks across jurisdictional boundaries (Free et al., 2019; Poloczanska et al., 2016; Young et al., 2019). These impacts have a range of implications for fisheries management (Pinsky et al., 2018). As transboundary fisheries become more common with climate change, fisheries can face access problems due to the relatively slow speed of negotiations and changes to management agreements (McIlgorm et al., 2010). Existing projections of marine biodiversity, global catch potential and fisheries revenues under climate change predict significant impacts, with biodiversity of marine species decreasing in the tropics (García Molinos et al., 2015), a re‐distribution of the catch potential globally (Cheung et al., 2010) and increases in revenues in northern latitudes as lower latitudes face reduction in profits (Lam, Cheung, Reygondeau, & Sumaila, 2016). These impacts are expected to affect heavily the people depending on marine resources for their livelihoods (e.g. Barange et al., 2018; Bell et al., 2013; Young et al., 2019).

Tuna fisheries are among the fisheries with greatest importance for economies and societies worldwide, targeting some of the world's commercially most valuable fish species. Tuna production increased from <0.6 million tons in 1950 to 7.7 million tons in 2014 (FAO, 2018). Among tuna species, tropical tunas, that is, Skipjack tuna (SKJ) *(Katsuwonus pelamis,* Scombridae), Yellowfin tuna (YFT) *(Thunnus albacares,* Scombridae) and Bigeye tuna (BET) *(Thunnus obesus,* Scombridae) account for the highest catches, making up for about 75% of tuna and tuna‐like global catches (FAO, 2018). These species are expected to be affected by anthropogenic climate change (Muhling et al., 2015), shifting their distribution, migration times, physiological rates and abundance with consequences for catchability to fisheries (Báez, Pascual‐Alayón, Ramos, & Abascal, 2018; Brill & Hobday, 2017). Due to the migratory nature of these species, some tuna fisheries operate over large spatial scales and multiple jurisdictions, utilizing fishing agreements to access EEZs, and with significant fishing activity in the high seas (Mullon et al., 2017). The importance of these fisheries for economies and livelihoods, together with the transboundary nature of the stocks and management, and the threat of climate change to tropical tuna species make it crucial to understand the challenges regarding future sustainability. Moreover, the interactions between management institutions, international agreements and climate‐driven changes in the tropical tuna fisheries need to be understood to plan effective adaptation to climate change.

Considerable effort has been expended on projecting tropical tuna fisheries activity under climate change scenarios (Asch, Cheung, & Reygondeau, 2018; Dell, Wilcox, Matear, Chamberlain, & Hobday, 2015; Lehodey et al., 2017; Michael, Wilcox, Tuck, Hobday, & Strutton, 2017; Yen, Su, Teemari, Lee, & Lu, 2016), but there is still little evidence showing impacts of climate change on the activity of fleets targeting tropical tunas (Monllor‐Hurtado, Pennino, & Sanchez‐Lizaso, 2017) or the consequences for dependent societies, economies and fisheries governance (Dueri et al., 2016; McIlgorm et al., 2010; Mullon et al., 2017). Regarding institutions, mainly two Regional Fisheries Management Organizations (RFMOs) take into account climate change considerations (i.e. the Inter‐American Tropical Tuna Commission and the Commission for the Conservation of Southern Bluefin Tuna; Gutierrez, 2016). The International Commission for the Conservation of Atlantic Tunas (ICCAT) is increasingly taking into consideration climate change, although mostly for the western Bluefin tuna stock assessment (Hobday et al., 2019). As with other food production sectors, integrating biophysical information together with social and economic factors is important when developing management response options to climate change.

Here, we investigate evidence of climate change affecting the recent distribution of tropical tuna fisheries using time series analyses of effort data. Then, we explore the role of technological and institutional actions associated with the distribution shifts, including the existing international agreements and management regulations. We focus on the east Atlantic Ocean, where a number of fleets operate. Our goal is to improve the understanding about how climate, management and governance interact in tropical tuna fisheries, with methods that are replicable and transferable.

## 2 MATERIALS AND METHODS

Effort data from the ICCAT and sea surface temperature anomaly (SSTA) data have been used to evaluate whether climate change is impacting the effort distribution of purse seine (PS) fisheries operating in the east Atlantic Ocean. An analysis of the spatial change in effort distribution is conducted using a centre of gravity (COG) approach. Empirical orthogonal functions (EOFs) are used to assess the spatiotemporal changes in effort anomaly (effortA) and SSTA. These functions generate spatial patterns and time series of effort and temperature that are correlated. Finally, random forest models are used to explore the influence of management, institutional agreements and technological changes on the effort. All analyses are performed using R software (version 3.5.1; R Core Team, 2018). R scripts for replication are available in GitHub/irrubio/tropituna_fishery_change (see the workflow of scripts in Figure S1).

### 2.1 Tropical tuna fisheries data

Monthly effort (fishing hours) and catch data of all PS fisheries targeting tropical tunas from 1991 to 2017 in the Atlantic Ocean were downloaded from the ICCAT website (ICCAT, 2018). This PS database is at a 1 by 1 degree resolution. For our analysis, PS data were limited to the eastern Atlantic Ocean (29°N, 30°S, 19°E, 35°W). Effort data were aggregated by summing to 5 by 5 degrees and also by quarter to keep the representativeness of the data in the study area for the EOF analysis. Then, effortA was calculated by subtracting the quarterly mean over the entire data period from the data (Benestad, Hanssen‐Bauer, & Chen, 2008; Bjornsson & Venegas, 1997). Quarterly effort was also calculated at 1 by 1 resolution by summing for the COG analysis.

In this study, we assume that the location of the reported fishing effort represents the distribution of the fleet activity (Davies, Mees, & Milner‐Gulland, 2014). Our main objective is to understand any fleet activity distribution response to climate change. Previous studies have considered catch and catch per unit effort (CPUE) spatial changes, using these variables as proxies for abundance. These studies are, however, not free from caveats, especially for PS fisheries (see in Kaplan et al., 2014 and Tidd, Brouwer, & Pilling, 2017). In a preliminary analysis, we found evidence of distributional changes in the fishery based on catch and CPUE data (calculated as the weight [*t*] caught by operation mode per fishing hour), but PS CPUE is not considered a good proxy for abundance (Kaplan et al., 2014; Maunder et al., 2006). To test for any relationship between catch/CPUE and abundance, we correlated estimated yearly biomass by the ICCAT Standing Committee on Research and Statistics (SCRS) with yearly catch/CPUE on fishing aggregation devices (FADs) and free schools of YFT and BET (SKJ biomass data are nowadays unavailable from the SCRS). Only YFT catch data on free schools were significantly correlated with abundance, as catches or CPUE on FADs did not follow (and were decoupled from) biomass trends (Figures S2 and S3); and BET correlations between catch/CPUE and estimated biomass were non‐significant. However, we were unable to use YFT catch location when fishing on free schools as a proxy for YFT distribution since the data were too patchy to allow the EOF analysis. For all these reasons, we conducted the final analysis with a focus on the distribution of fishing effort.

### 2.2 Sea surface temperature anomaly data

**Figure 1:**
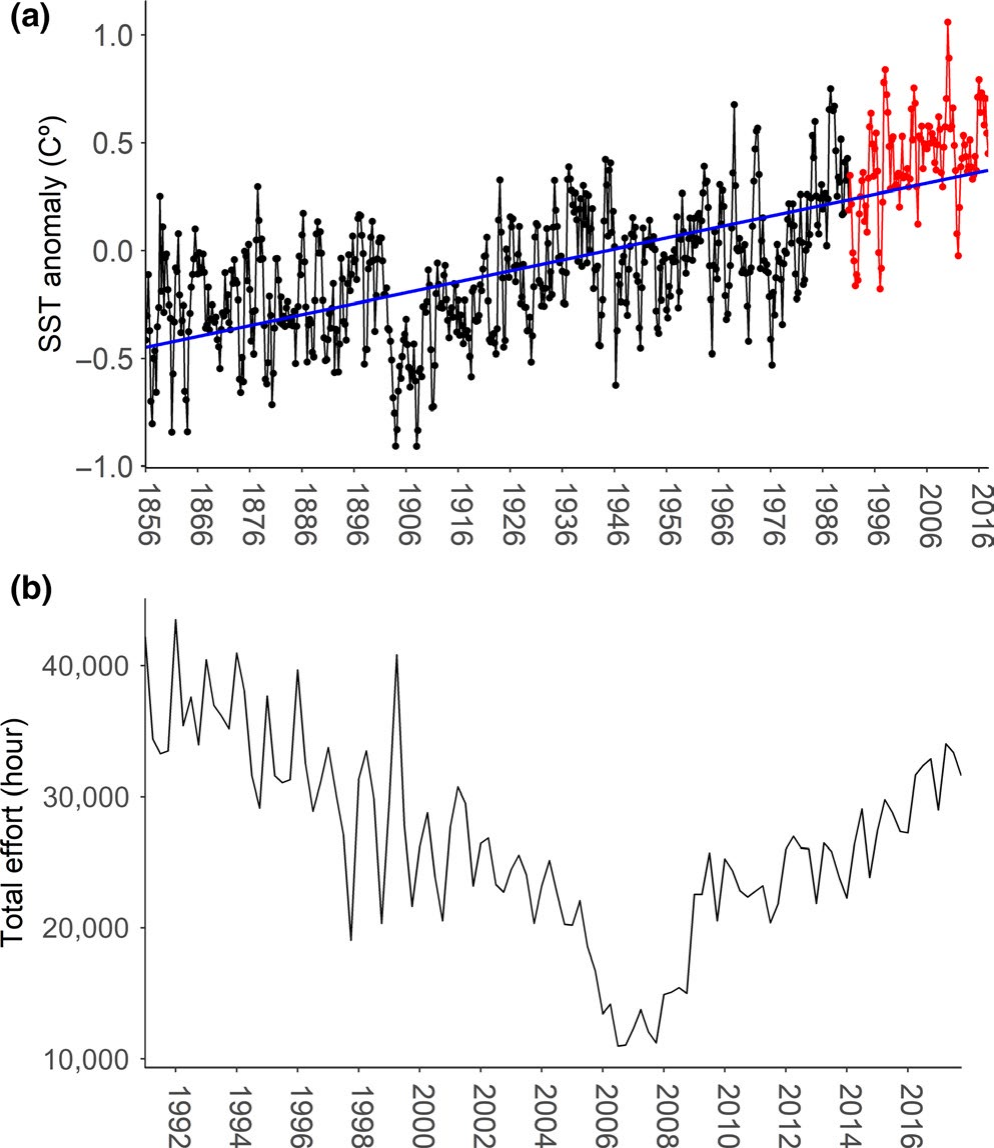
Sea surface temperature anomaly (SSTA) in the study area from 1856 to 2017 (a) and total effort in fishing hours by all purse seine (PS) fleets in the study area from 1991 to 2017 (b). SST anomalies are based on the 1951–1980 time period. The blue line represents a linear regression fitted to the SSTA data (*p* < .05). The red line represents SSTA in the study period 1991–2017 (see a detailed view of SSTA data for 1991–2017 in Figure S5) [Colour figure can be viewed at wileyonlinelibrary.com]

To describe environmental change, we used monthly Kaplan SST V2 anomaly data from 1856 to 2017 with a spatial resolution of 5 by 5 degrees (NOAA/OAR/ESRL.PSD, 2018), which were aggregated by quarter by averaging for the study period and area. These SST anomalies are based on the 1951–1980 time period. We used 5 by 5 degree resolution to match effort resolution for the EOF analysis. While this coarse resolution does not permit analysis of environmental features (e.g. eddies or fronts), we are seeking to understand large scale shifts linked to highly mobile fleets targeting highly migratory species. We used the data from 1856 to 2017 to show the long‐term change in SSTA in the study region (Figure 1); however, for the rest of the analysis, we matched SSTA and effort data temporal scales, that is 1991–2017.

### 2.3 Institutional and technological data

In order to investigate the role of technological and institutional actions in the effortA distribution shifts, we collected information on PS institutional arrangements, management regulations and technological change in the study region over the period 1991–2017. The European Union has public Sustainable Fisheries Partnership Agreements (SFPAs) with African countries that allow European vessels to enter EEZs of those countries. These agreements could have had an impact on the fishery distribution since European PS represents 56% of the PS tropical tuna catch in the study area (calculated from the database used in 2.1). We focused on two variables, the number of vessels allowed to enter a specific EEZ by SFPA and the total number of SFPAs in the region by quarter. Data until 2012 were obtained from Le Manach et al. (2013), and for 2012 to 2017 from the web‐based sources and agreements/protocols of European databases (European Commission, n.d.; European Union, n.d.). Unfortunately, information on private agreements is not available publicly and we were not able to include it in the analysis.

The ICCAT is the management body that establishes fishing regulations, including Total Allowable Catches (TACs) that apply in the region. We compiled information on the different management and conservation policies taken by the ICCAT to preserve tuna fisheries in good status (not overfished). Management actions by quarter were extracted from ICCAT official recommendations available on the website (ICCAT, n.d.), which include information on TACs when set (i.e. presence or absence of a TAC as a binomial variable), as well as fishing time–area closures (presence or absence of a closure as a binomial variable).

Tropical tunas are targeted by some fleets that employ advanced technologies including FADs with echo‐sounder buoys. These fleets have modernized substantially over the time period considered, and changes in technology may impact effort. To represent FAD fishing patterns, that is technological change, catch proportion on FADs by quarter (%, all species included) was calculated between 1991 and 2017, using the ICCAT database from 2.1.

### 2.4 Distribution change analysis

To capture the spatial patterns of the PS tropical tuna fishery and to investigate year‐to‐year variations, the latitudinal COG of the effort is calculated every year. The COG represents the mean location of the effort (Saraux et al., 2014). We consider annual COG north and south of the equator separately to check for poleward expansion in each hemisphere. The COG is calculated using Equation (1),

(1) $$\text{COG}=\frac{\sum_{i=1}^{n}{\text{latitude}}_{i}*{\text{effort}}_{i}}{\sum_{i=1}^{n}{\text{effort}}_{i}},$$

where *n* is the number of fishing sets, effort*_i_* is the effort in the *i*th set and latitude*_i_* is the latitude of the *i*th set. A linear regression is used to evaluate trends over time in COG in each hemisphere, and COG is also correlated (Pearson) to SSTA.

### 2.5 Temperature and effort distribution analysis

To further assess the variability of the tropical tuna fishery distribution over time and its relationship with SSTA, we apply empirical orthogonal functions (EOFs) to the quarterly PS effort anomaly data (effortA) (Bjornsson & Venegas, 1997; Preisendorfer, 1988) and the quarterly SSTA. We use the “sinkr” package in R (version 0.6; Taylor, 2017). The EOFs are found by computing the eigenvalues and eigenvectors of the effortA or SSTA covariance matrix. The derived eigenvalues provide a measure of the per cent variance explained by each mode of variability. Then, the most informative EOFs are identified, and temporal and spatial structures investigated. However, the variance of effortA or SSTA is not equal at all gridpoints; therefore, the “local” explained variance (LC) is also calculated using Equation (2).

(2) $$\text{LC}=\frac{e_{\mathrm{ji}}^{2}\lambda_{i}}{\sum_{k=1}^{k_{\max}}\lambda_{k}e_{\mathrm{jk}}^{2}},$$

which is equivalent to the fraction of variance expressed by the *i*th EOF at each *j*th grid point over the total variability reconstructed by the leading *k*_max_ EOF (Ganzedo, Alvera‐Azcárate, Esnaola, Ezcurra, & Sáenz, 2011). *λ_i_* represents the eigenvalue of the covariance matrix associated with the *i*th eigenvector *e_i_*. This analysis does not allow for missing data, thus, effortA is reconstructed by means of the Data Interpolating Empirical Orthogonal Functions method (DINEOF) (Beckers, Barth, & Alvera‐Azcárate, 2006), which has already been applied to the ICCAT dataset and validated by Ganzedo, Erdaide, Trujillo‐Santana, Alvera‐Azcárate, and Castro (2013). Spatiotemporal time series with <25% of missing data were selected for reconstruction (Ganzedo et al., 2013). In total, the dataset for EOF analysis has 26 spatial series each with 108 time values (quarters from 1991 to 2017).

The advantage of performing an EOF analysis is that a small number of leading EOF can explain a large fraction of the total variance of the whole dataset, as other studies applied to fish ecology and fisheries science have found (Marshall et al., 2016; Petitgas, Doray, Huret, Masse, & Woillez, 2014; Saraux et al., 2014). Then, posterior testing can be done to see whether a relationship exists between effortA and SSTA by performing Pearson correlations between the EOF temporal structures of effortA versus SSTA. This methodology takes into account the entire spatiotemporal structure of the datasets (including latitude and longitude) which is not possible with the previous COG analysis.

### 2.6 Institutional and technological analysis

Two random forest models are used to evaluate the influence of management, international agreements and technology on the effort and the temporal structure resulting from the EOF analysis of the effortA compared with other variables (e.g. SSTA) using the “randomForest” package in R (version 4.6‐14; Liaw & Wiener, 2002). This method has been previously applied in fisheries research (e.g. Melnychuk, Banobi, & Hilborn, 2013; Pons et al., 2017) and allows for non‐linear relationships between predictors and a response variable without making any parametric assumptions about the distribution of the response variable. Random forests (Breiman, 2001) are an ensemble method, which build a selected number of regression trees (*m*) from a boostrap sample of the original data set. Kuhn and Johnson (2013) suggest using at least 1,000 trees that in our case are adequate to stabilize the mean squared error (*MSE*) of the model. For each regression tree, a set of predictors *(mtry)* are randomly selected from the original predictors at a given node. We use the default value of *mtry,* which is equal to a third of the predictor variables (Liaw & Wiener, 2002). The best predictor is then determined, and the data split in two groups such that the overall sum of squared errors is minimized (Kuhn & Johnson, 2013). This process continues until a tree is built, and multiple predictor variables can be shown to influence the response variable. We present from the analysis a variable importance plot for visualizing the percentage of increase in the *MSE* (%IncMSE) when variables are randomly permuted, which gives a measure of how influential each considered predictor variable is on predicting the response variable. We also include the results of partial dependence plots that provide insight into the directionality of the effect for a given predictor (Berk, 2008). Before conducting random forest, predictors were tested for collinearity using generalized variance inflation factors (Fox & Monette, 1992), which were <6.0 suggesting little possibility of confounding among the predictor variables (Zuur, Ieno, & Elphick, 2010).

In our two random forest models, the response variables are the quarterly effort in fishing hours and the quarterly temporal structure (PC1) resulting from the EOF analysis of effortA (effort: continuous, mean ± *SD* [26,559 ± 7,361], range [10,972–43,494]; PC1 effortA: continuous, mean ± *SD* [0.0 ± 2.2], range [−5.7 to 6.7]). The predictors are listed and described in Table 1.

**Table 1:** Predictor variables of the random forest models, note that the response variables are the effort and the quarterly PC1 of effort anomaly resulting from the empirical orthogonal function analysis

Predictor	Description	Mean ± *SD*	Range (min–max)
SSTA	Continuous. The quarterly sea surface temperature anomaly mean in degrees	0.4 ± 0.2	−0.2 to 1.1
quarter	Factor. Quarter when the data were recorded. 1: January to March; 2: April to June; 3: July to September, 4: October to December	–	1–4
FAD_prop	Continuous. The quarterly proportion of total catch (all species included) on FADs representing the major technological changes in %	62.5 ± 15.8	25.9–91.7
TAC	Binomial factor. Is there a total allowable catch established? Yes (1)/ no (0)	–	0–1
closure	Binomial factor. Is there time‐area closure established? Yes (1)/ no (0)	–	0–1
agr_num	Continuous. Agreement number, number of Sustainable Fisheries Partnership Agreements (SFPAs) in place by quarter	8 ± 2	4–10
agr_vessel	Continuous. Vessel number allowed by SFPAs in place by quarter	31 ± 4	25–41

## 3 RESULTS

**Figure 2:**
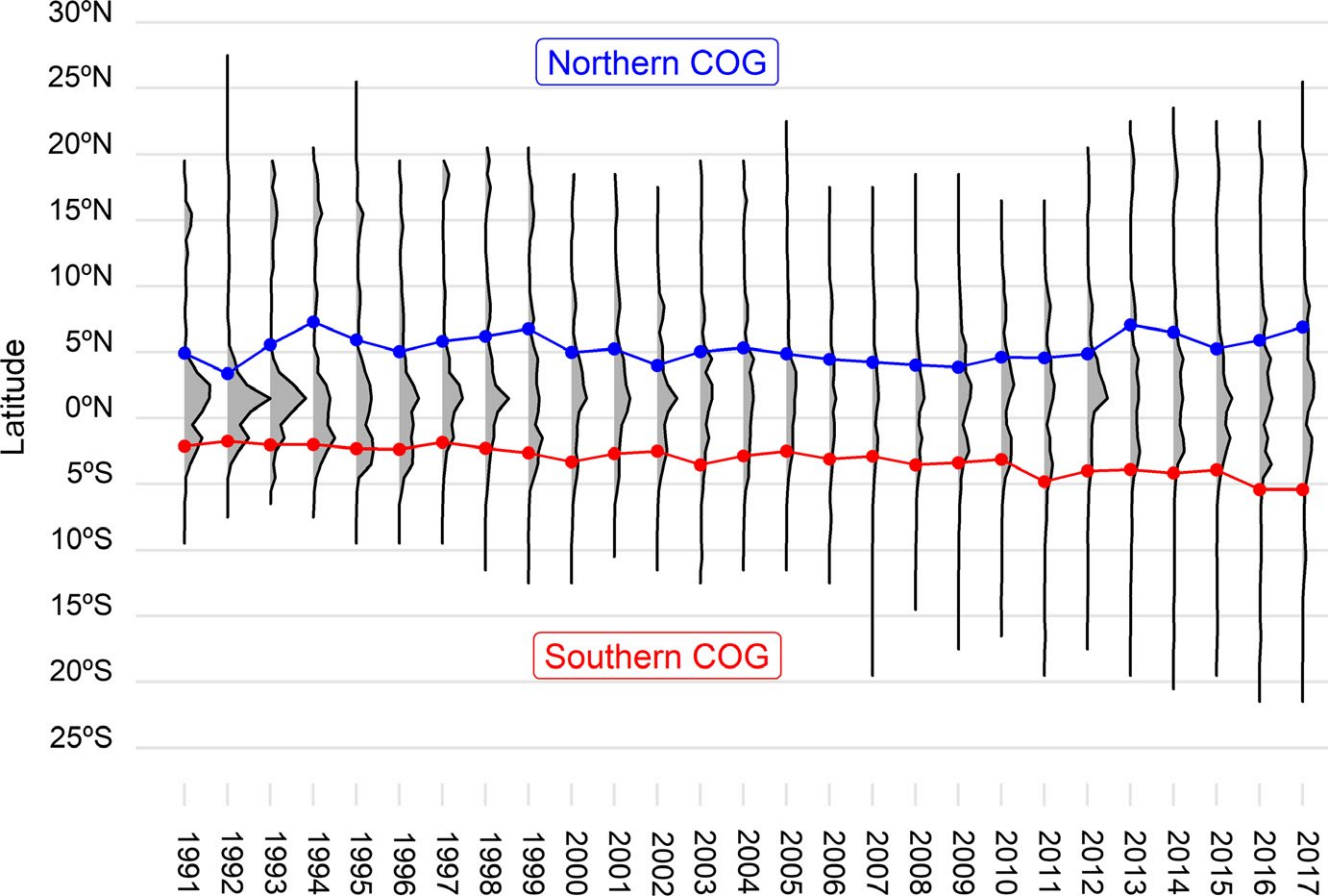
Latitudinal north (blue line‐dots) and south (red line‐dots) centre of gravity (COG) changes of effort from 1991 to 2017. Grey shading represents the annual effort distribution by latitude [Colour figure can be viewed at wileyonlinelibrary.com]

Records of SSTA in the study region show a statistically significant increasing trend over the period 1856 to 2017 (Figure 1a), where SSTA has increased by 0.82°C. Total effort progressively decreases after 1999 but increases again after 2009 (Figure 1b). Changes in the distribution of the fishery over time only follow a clear pattern for the southern COG of effort (Figure 2), which significantly shifts towards southern latitudes from the equator between 1991 and 2017 (*p* < .05) and correlates with SSTA with a Pearson r coefficient of −0.3 (*p* < .05). When the SSTA is higher, the COG shifts south. The northern COG of effort does not follow any clear distribution trend over the study period and area (Figure 2) and does not correlate with SSTA.

**Figure 3:**
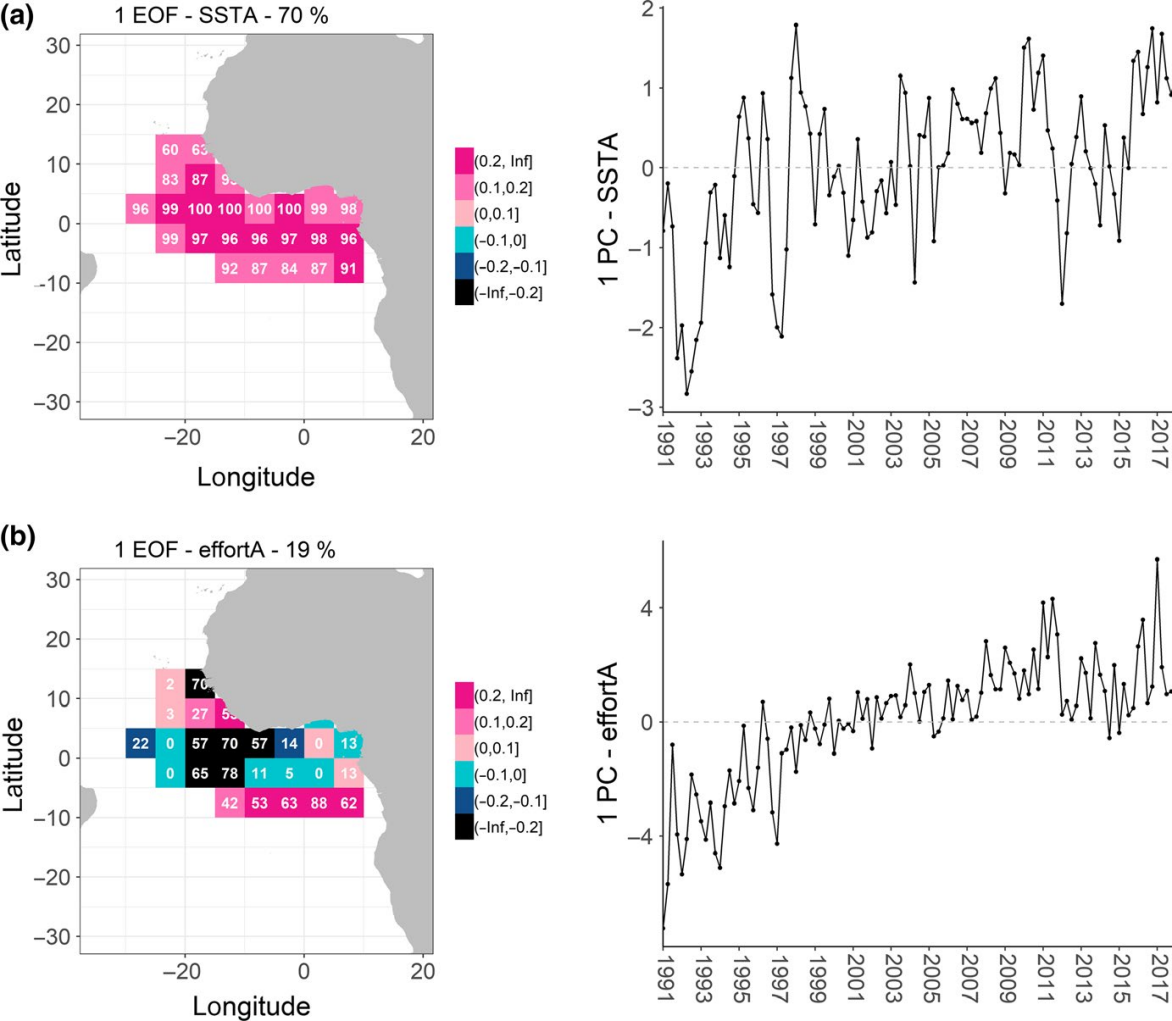
Empirical orthogonal function (EOF) results for sea surface temperature anomaly (SSTA) (a) and effortA (b). Left: spatial structures of the EOF. Total variance (%) appears in the title and the “local” explained variance (%) inside pixels. Right: temporal structures (PCs) of the EOF [Colour figure can be viewed at wileyonlinelibrary.com]

In order to further explore changes in distribution and any relationship with temperature, we performed an EOF analysis on SSTA and effortA and then examined the correlation between the resulting temporal structures (Figure 3; see uncorrelated structures of second and third leading EOF in Figure S4). The first leading EOF of the SSTA accounts for 70% of the data variance and the first leading EOF of the effortA for 19% (Figure 3). Correlation between the EOF temporal structure of SSTA and effortA is significant (*p* < .05), with a Pearson *r* coefficient of 0.5.

Examination of the “local” explained variances shows that the highest reconstructed local explained variance of the first temperature anomaly EOF is concentrated in equatorial waters of the study area (Figure 3a). The spatial structure is quite homogeneous since all waters behave in a similar way (pink colour, Figure 3a). For years when the temporal structure or principal component (PC) is positive (e.g. 2016–2017 in Figure 3a), warming is observed in the whole study area, particularly in waters closer to the equator. In addition, the first EOF of effortA shows a different spatial pattern (Figure 3b). In general, the effortA in central west equatorial waters and waters off Senegal behaves in the opposite way to the effortA in southern waters and waters between 5° and 10°N. This means that when the PC of effortA is positive (e.g. 2016–2017 in Figure 3b), effort increases in the pink‐pixel waters and decreases in the black‐pixel waters (Figure 3b). Pixels must be interpreted with caution due to large variation in local explained variance (e.g. range 0%–88%). If we spatially link SSTA to effortA, which correlate positively for the temporal EOF structure, effort shifts from central west equatorial waters and waters in front of Senegal towards southern waters of the study area and waters between 5° and 10°N when the PC of SSTA is positive and vice‐versa. Therefore, with warmer waters (e.g. as a result of climate change), effort shifts southeastwards from central west equatorial waters of the study area and towards waters between parallel 5° and 10° north latitude.

**Figure 4:**
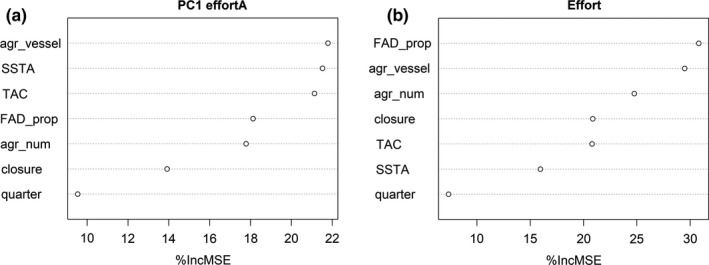
Variable importance score of different predictors on the PC1 effortA from the empirical orthogonal function (EOF) analysis (a) and effort (b). The most influential variables are those with the greatest percentage increase in the mean squared error (%IncMSE)

**Figure 5:**
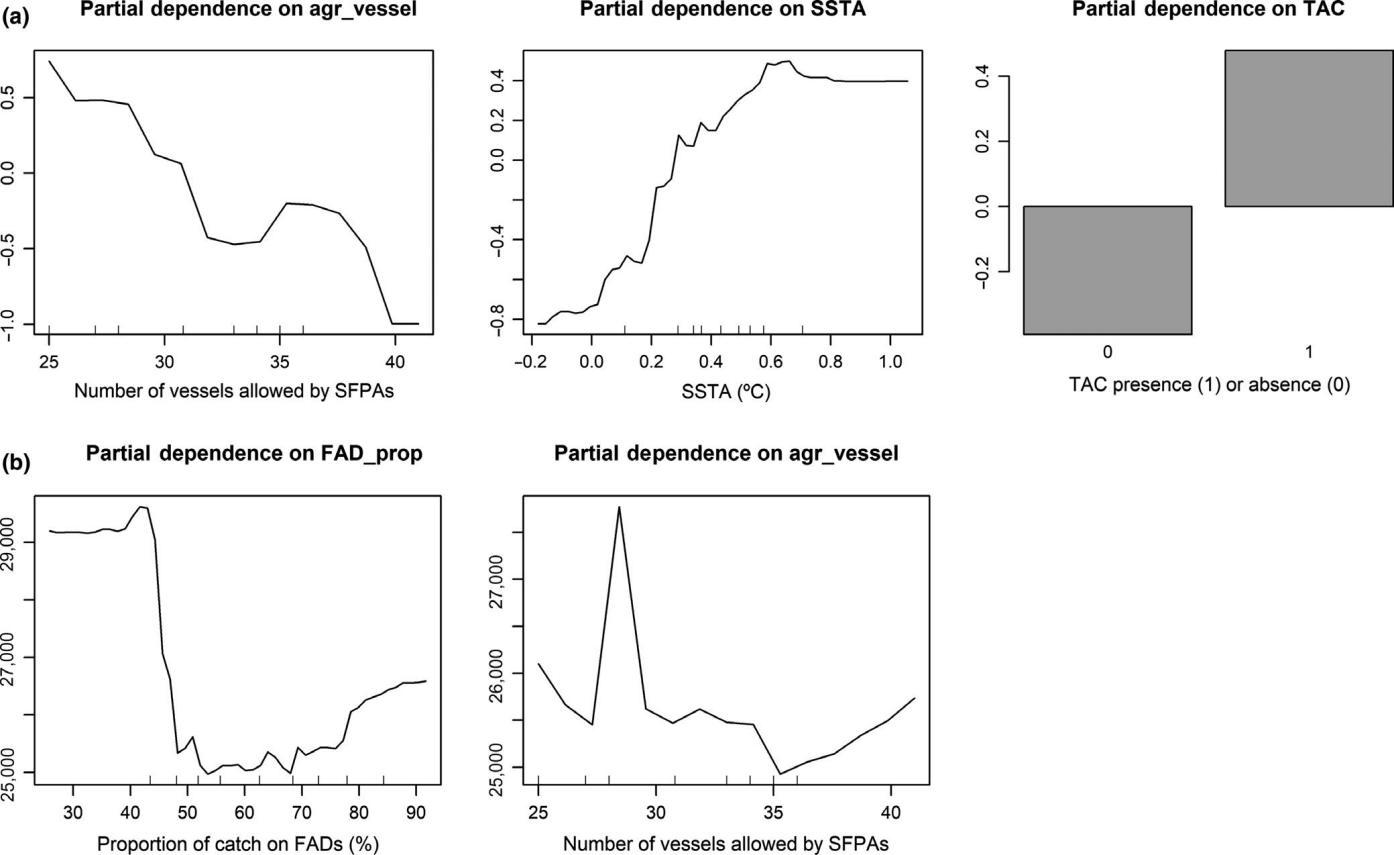
Partial dependence plots of the most important predictors of PC1 effortA from the empirical orthogonal function (EOF) analysis (a) and effort (b). The vertical axis is the conditional mean of the response (PC1 effortA or effort) for different values of the predictor in question, with all other variables fixed

A relationship between the first component of the temporal structures (PC1) of effortA and SSTA has been found by means of the EOF analysis and posterior correlation, which partially explains spatiotemporal shifts of the PS fishery. The southern COG of effort also correlates with SSTA. To evaluate the influence that management, institutional agreements and technology have on the trends of the PC1 effortA and effort compared with other variables (e.g. SSTA), a random forest model was fitted to the PC1 effortA explaining 59% of the data variance, as well as to effort, explaining 60% of the data variance. The variables with the largest percentage of increase in the mean squared error are the most important predictors, which are the number of vessels allowed by SFPAs (agr_vessel), SSTA and the presence/absence of a TAC in the case of PC1 effortA (Figure 4a) and technology represented by the proportion of catch on FADs and agr_vessel in the case of effort (Figure 4b). The partial dependence plots for the most important predictors of PC1 effortA (Figure 5a) and effort (Figure 5b) show that PC1 effortA is positive with a higher number of vessels allowed to access certain EEZs, with higher SSTA and with the presence of a TAC. Effort is higher with the smallest and highest proportions of catch on FADs, while there is no clear trend from the partial dependence plot on the vessel number allowed by agreements (agr_vessel). While effort appears to be higher with the smallest and highest proportions of catch on FADs, this pattern is driven by very few data points and may not traduce the overall pattern in the relationship between effort and proportion of catch on FADs.

## 4 DISCUSSION

PS fisheries activity in the central east Atlantic Ocean has shifted southward from the equator from 1991 to 2017. However, the northern distribution of the fleet activity does not follow a clear pattern, but tends to move towards waters between 5° and 10°N. By focusing on catch compositions, Monllor‐Hurtado et al. (2017) reported a shift of tuna catches in sub‐tropical latitudes on a global scale from 1965 to 2011 by investigating the percentage of tropical tuna in longline catches. They concluded that at least in the Atlantic and Indian Oceans, tropical tuna catches have reduced in tropical areas and they attribute these changes to ocean warming. Cheung, Watson, and Pauly (2013) also showed ocean warming has already affected global fisheries, with an increasing dominance of catches of warmer waters species at higher latitudes. Our analyses showed a relationship between spatiotemporal changes in effort distribution of purse seiners targeting tropical tunas and SSTA in the study area, meaning that the fleet activity is being affected by climate change. In addition to temperature, other environmental conditions might be influencing the spatial distribution of the fleet, for example upwelling systems in the study area that are expected to decline with climate change with unknown consequences to fishing communities (Sylla, Mignot, Capet, & Gaye, 2019). If we link the response of the fleet to what is being observed on tropical tuna species, according to Erauskin‐Extramiana et al. (2019), tuna habitat distribution limits are already shifting poleward (on average, 6.5 km per decade in the northern hemisphere and 5.5 km per decade in the southern hemisphere). It is thus consistent to see a southward movement of the fleet over time partially explained by climate change as a response of an ecological shift. However, we did not see a clear northward trend from the equator, suggesting other factors are involved. Indeed, the scale of the habitat spatial shift observed by Erauskin‐Extramiana et al. (2019) is smaller than the one of the fleet during the study period, reaffirming that other variables (e.g. management or technological variables) are also playing a role in the distribution of the fishery.

Our results show that the change in distribution of the fishery is related to a combination of environmental (SSTA) and human factors. The EOF analysis has isolated the spatiotemporal distribution patterns of effort that are linked to SSTA; however, other variables seem to have a higher effect on global effort such as the proxy we used for technology advances. Changes in technology are crucial and affect effort (Tidd et al., 2017). Since the mid‐1990s, FADs have become an important means by which purse seiners catch tropical tunas, and the PS fishery is now mostly dependent on FADs in the Atlantic Ocean (Maufroy et al., 2017). Throughout the 2000s, several other technological improvements occurred in the fishery, including the use of radio buoys with GPS system to locate FADs and the introduction of echo‐sounder buoys to monitor the amount of biomass aggregated under FADs (Lopez et al., 2014). There has been an associated shift in fishing strategies; before the FAD revolution, successful sets on free schools required a deep understanding and knowledge of the environment and target species acquired over many years of fishing experience. Now, skippers rely on locating FADs and using new technologies for successful sets; they have become technology managers and tuna harvesters following FADs (Gabantxo Uriagereka, 2003). With regard to effort, FAD utilization was the most important variable explaining effort during the study period, a crucial factor to include when studying effort changes of purse seiners in the Atlantic Ocean.

Furthermore, the role of institutions is important in limiting the number of vessels allowed to fish in EEZs through public SFPAs for example, however, certain vessels not having access to EEZs can still fish in the high seas or access these EEZs through private agreements (no information on private agreements was available to include in our analysis). Results indicate that public SFPAs influence overall spatiotemporal changes of effort in the region and have a higher role when explaining effort than SSTA. This suggests that human management has the potential of overtaking distribution impacts caused by climate change on fisheries activity (Barange, 2019; Gaines et al., 2018). The management institutions are RFMOs, that is the ICCAT in our study area, and seek to ensure the sustainable use of fishing resources (Cullis‐Suzuki & Pauly, 2010). There has recently been an effort to establish a FAD management plan and limit the number of FADs, which is currently set to no more than 500 FADs with or without instrumental buoys active at any time per vessel in the Atlantic Ocean (ICCAT, 2016). Management actions such as setting TACs and spatial closures also took place in the study period, being the presence of a TAC influential on the spatiotemporal distribution of effort and the presence of a closure similar to the presence of a TAC when influencing overall effort. Although climate change is posing an additional consideration for fisheries management, it is not yet taken into account by the ICCAT. Fortunately, according to Pentz, Klenk, Ogle, and Fisher (2018), the ICCAT does have a relatively high potential capacity to manage high seas resources effectively under climate change. While many institutional actions represent an indirect set of adaptations to climate change in that they all seek the sustainability of tropical tunas, direct and specific climate‐related actions are needed to avoid undesirable socioeconomic consequences (Grafton, 2010; Leith et al., 2014).

Some models predict a biomass increase of Skipjack and YFTs in tropical areas as well as in most coastal countries’ EEZs in the long term, a decrease of BET (Dueri, Bopp, & Maury, 2014; Erauskin‐Extramiana et al., 2019) and a re‐distribution in both depth and horizontal distribution of tropical tunas (Deary et al., 2015; Dell et al., 2015; Erauskin‐Extramiana et al., 2019; Evans et al., 2015). Here, we did not take into account changes in the depth of fishing effort, but PS fishing may be more affected than other methods such as longline fisheries that can set hooks at greater depths (Marsac, 2017). Regarding latitudinal shifts, PS vessels in the east Atlantic Ocean are already going further south and north from the equator due to technological advances among other factors, but this also leads to increased fuel costs (see also Michael et al., 2017). Thus, home port location could become limiting if species shift further away. Other implications of shifting species concern the distribution of tropical tunas in EEZs of the coastal countries (Báez et al., 2018). Some countries in the Guinean Gulf might be losing resources and others gaining, which adds a challenge for the sustainability of the resources (Bell et al., 2013; Dubik et al., 2019; Pinsky et al., 2018). New public or private agreements and conflicts (e.g. Spijkers & Boonstra, 2017) might also arise between countries from tropical tunas’ re‐distribution.

The IPCC (2019) highlights the need to develop adaptation plans to minimize the effect of ocean warming on dependent communities, particularly in tropical regions which are expected to be more affected by climate change. Even if ecological impacts are expressed most strongly in tropical regions, dependent communities are also outside these regions. Therefore, it is crucial to analyse fishing‐dependent societies’ vulnerability in the face of climate change (e.g. Badjeck, Allison, Halls, & Dulvy, 2010; Colburn et al., 2016; Hobday et al., 2016; Young et al., 2019), distinguishing who is benefiting from fishing or has stopped benefiting, where and whether it is under a subsistence economy or free market economy and considering interactions between both.

Finally, identifying impacts of climate change on tropical tunas and their associated fisheries and management is of great importance to determine whether it will affect societies dependent on these resources. The method developed here to explore the effect of ocean temperature on the effort of tropical tunas in the east Atlantic Ocean over the period 1991–2017 showed that PS fisheries have shifted southward from the equator, which can be explained by climate change, institutional, management and technological factors. However, our results suggest that management can be a powerful tool to overcome climate change distribution impacts on fisheries activity. As fisheries management organizations have a crucial role to maintain resource sustainability, adaptation to climate change needs to be incorporated in their agendas, which must span environmental, institutional and socioeconomic considerations.

## Data Availability

The data that support the findings of this study are openly available in “tropituna_fishery_change” at http://github/irrubio/tropituna_fishery_change and https://doi.org/10.5281/zenodo.3574095.
