# Supplementary material for: Southward re‐distribution of tropical tuna fisheries activity can be explained by technological and management change
Source: Fish Fish (Oxf). 2020 Jan 28;21(3):511–21. doi: 10.1111/faf.12443 (PMC7317860; doi:10.1111/faf.12443)
Supplement: Supplementary file 1 [file FAF-21-511-s001.docx]

**Supplementary Information**

All the analyses performed in this article are reproducible through the scripts found in GitHub/irrubio/tropituna_fishery_change. These scripts follow a linear workflow from scripts starting by 1 to 5 (Figure 1):

1. Scripts starting with a 1. They correspond to the ICCAT catch and effort data preparation and the EOF analysis of effortA (article Figure 3).
2. Scripts starting with a 2. They correspond to SSTA data preparation for temporal evolution analysis (also for effort evolution analysis, article Figure 1) and EOF analysis of SSTA (article Figure 3).
3. Script number 3 correlates temporal structures of effortA and SSTA from EOF analysis.
4. Scripts starting with a 4 produce COG analysis (article Figure 2).
5. Scripts starting with a 5 produce random forest analysis (article Figures 4 and 5).


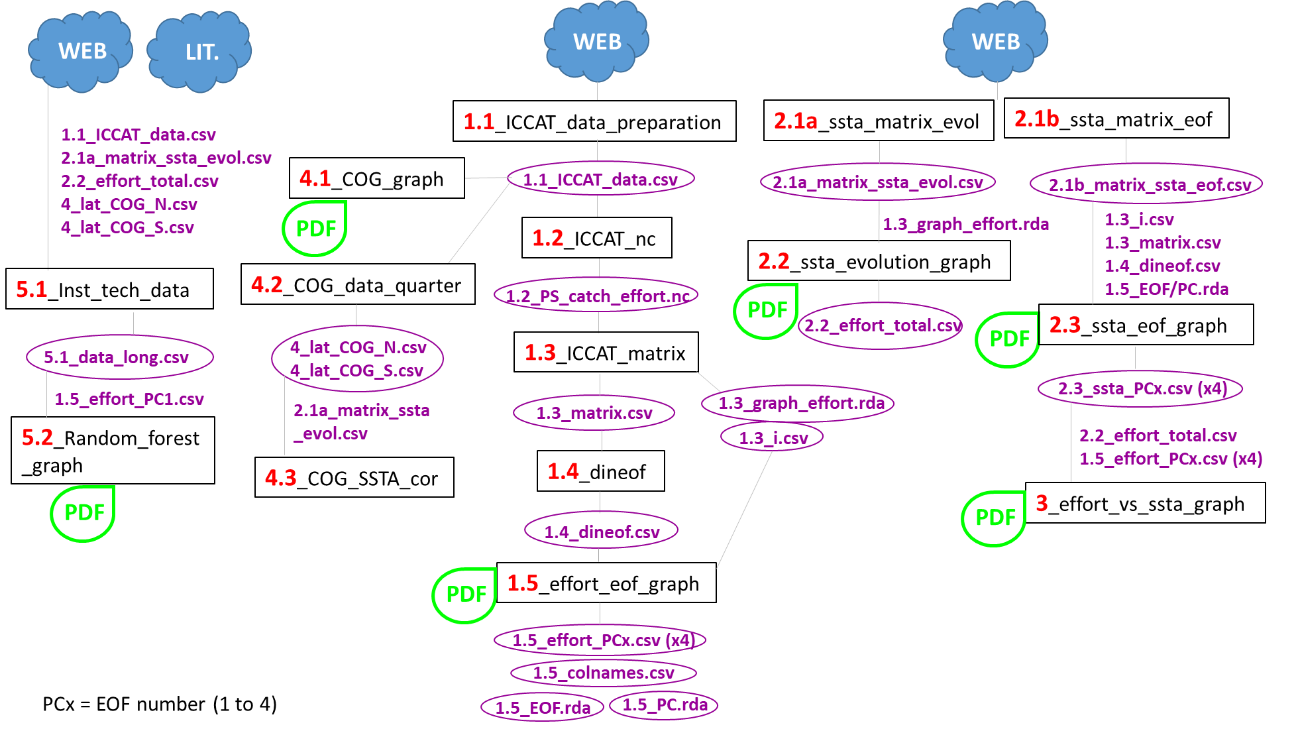


**SI Figure 1. Scripts workflow.** Script titles appear inside black boxes, data created in each script and needed for the next script appear in purple and green PDF bubbles indicate that the script produces Figures included in this article. Blue bubbles indicate the source of the data used (web or literature). Number of files produced that are higher than 1 appear inside brackets.


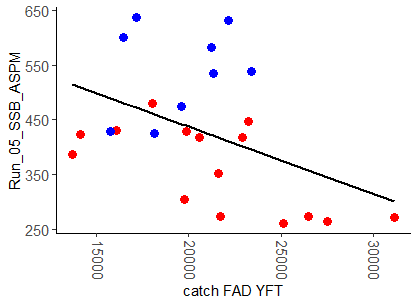

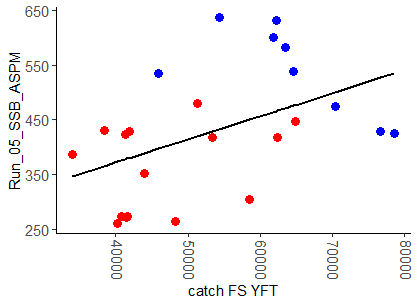


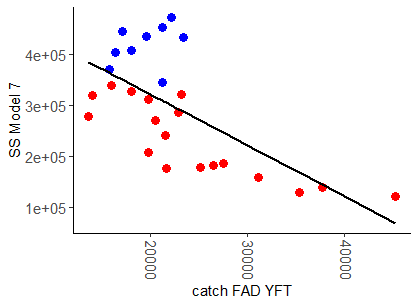

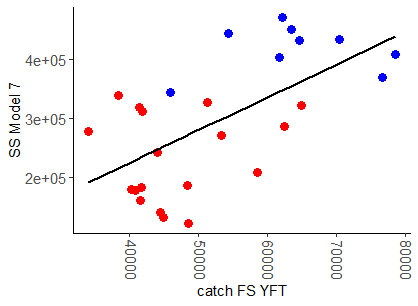


**SI Figure 2.** A significant linear relationship between catch (x-axis) and estimated biomass (y-axis) was only found for YFT when fishing on FAD and free schools (not for BET, unknown for SKJ). Blue: 1991-2000; red: 2001-2014**.**


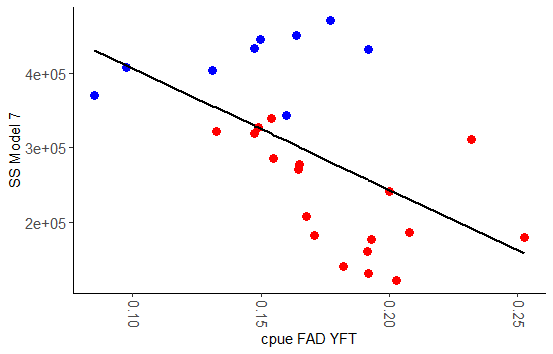

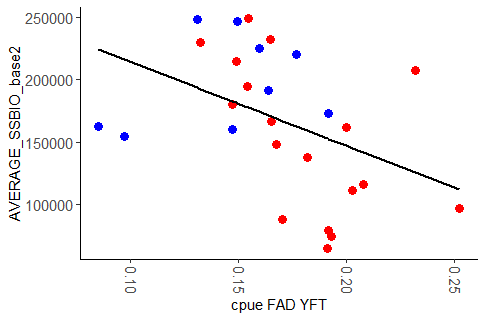

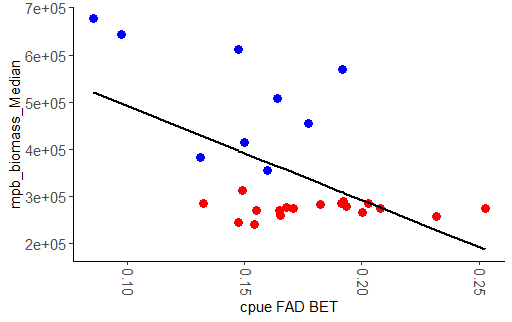

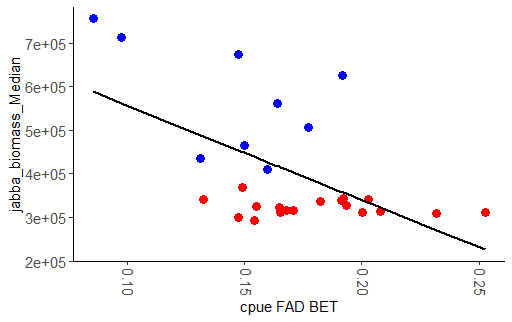

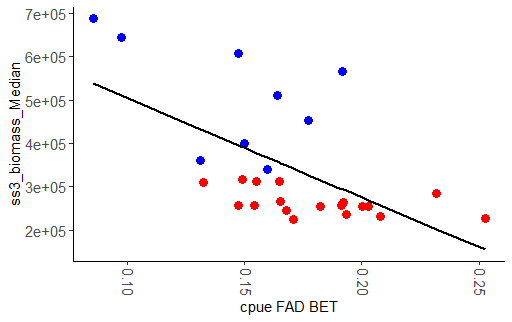


**SI Figure 3.** A significant linear relationship between CPUE (t/h; x-axis) and biomass (y-axis) was only found for YFT and BET when fishing on FAD. CPUE seems decoupled from biomass. Blue: 1991-2000; red: 2001-2014**.**


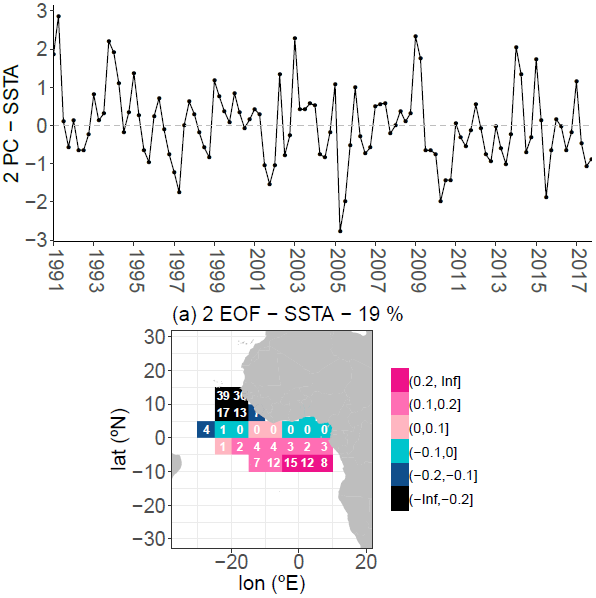

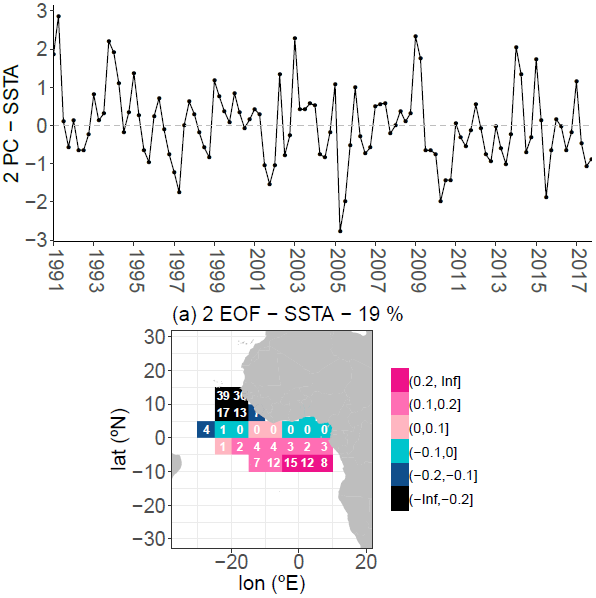


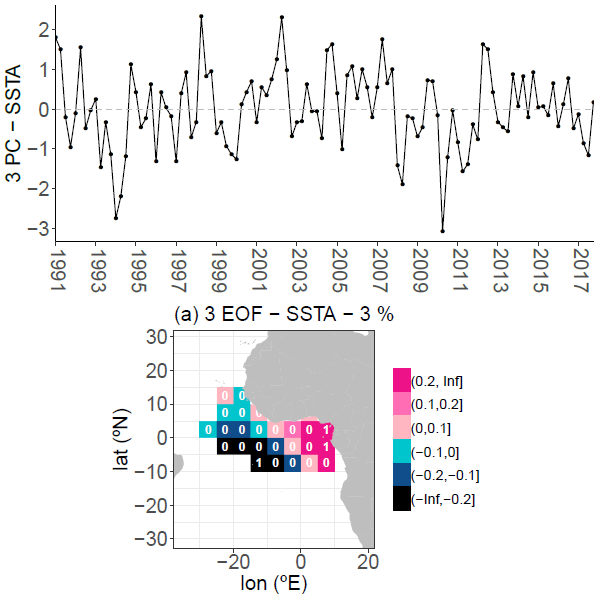

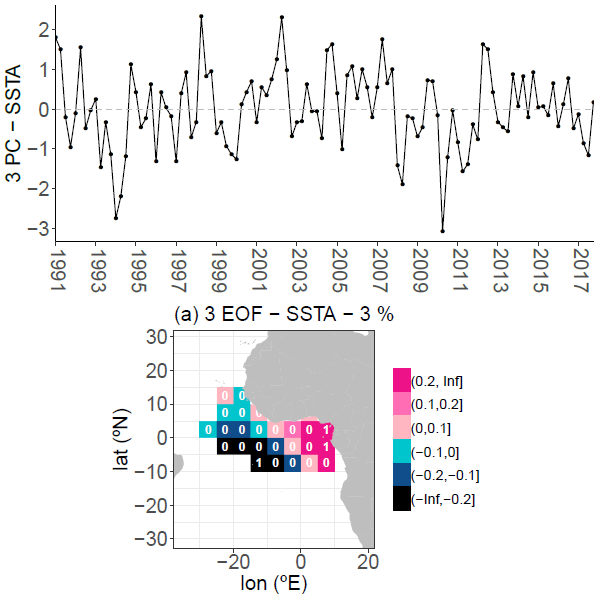


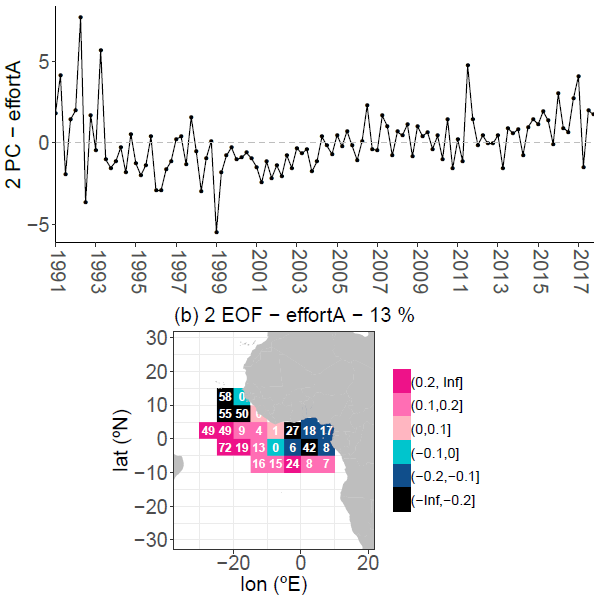

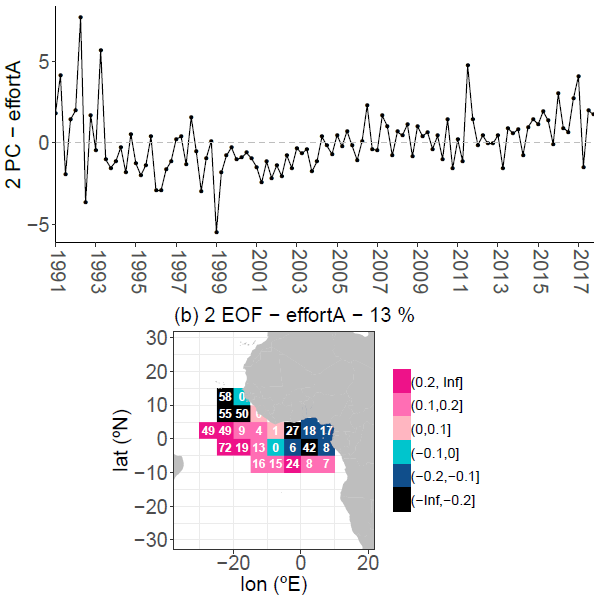


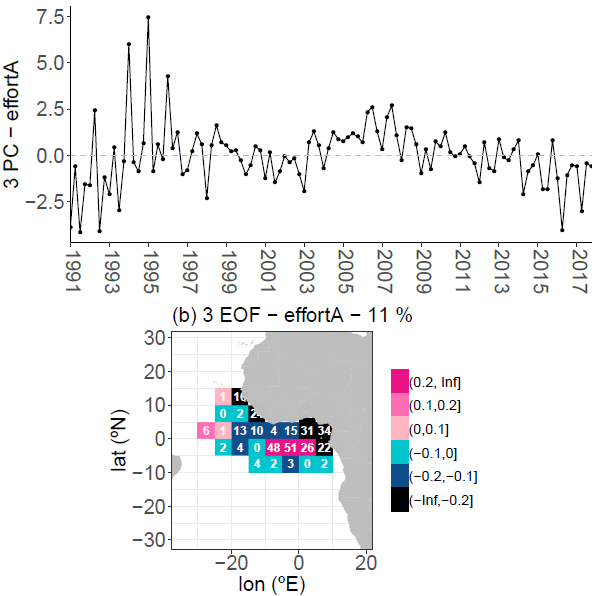

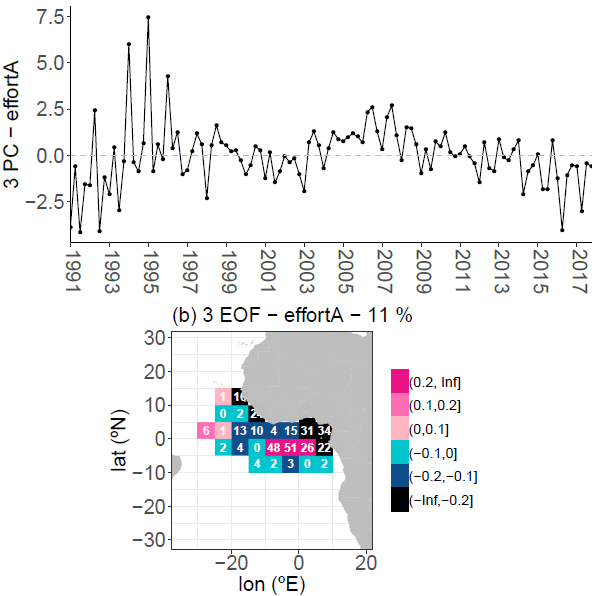


**SI Figure 4.** EOF uncorrelated results for SSTA and effortA. Left: spatial structures of the EOFs. Total variance (%) appears in the title and the “local” explained variance (%) inside pixels. Right: temporal structures (PCs) of the EOFs.

**
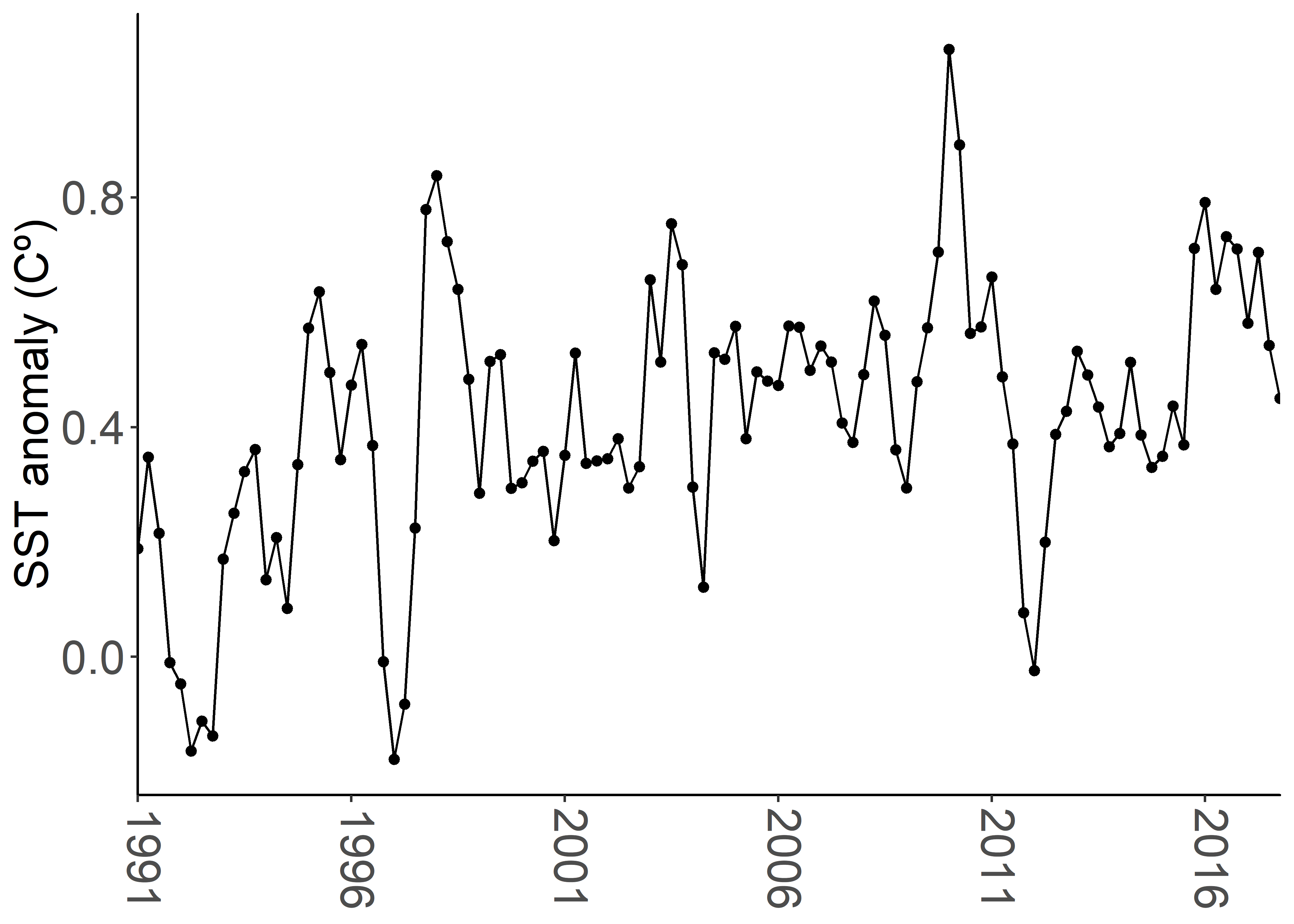
**

**SI Figure 5.** Sea surface temperature anomaly in the study area from 1991 to 2017.
